# RETRACTION: Impacts of Continuous Quality Improvement on Wound Pain in the Puncture Site of Arteriovenous Fistula in Haemodialysis Patient

**DOI:** 10.1111/iwj.70445

**Published:** 2025-03-26

## Abstract

**RETRACTION:** LiH. , LiuX.‐L. , HuangS.‐F. , and WenY.‐J. , “Impacts of Continuous Quality Improvement on Wound Pain in the Puncture Site of Arteriovenous Fistula in Haemodialysis Patient,” International Wound Journal 21, no. 3 (2024): e14697, 10.1111/iwj.14697.38468432 PMC10928246

The above article, published online on 11 March 2024, in Wiley Online Library (http://onlinelibrary.wiley.com/), has been retracted by agreement between the journal Editor in Chief, Professor Keith Harding; and John Wiley & Sons Ltd. Following an investigation by the publisher, all parties have concluded that this article was accepted solely on the basis of a compromised peer review process. The editors have therefore decided to retract the article. The authors did not respond to our notice regarding the retraction.

**RETRACTION:** H. Li , X.‐L. Liu , S.‐F. Huang , and Y.‐J. Wen , “Impacts of Continuous Quality Improvement on Wound Pain in the Puncture Site of Arteriovenous Fistula in Haemodialysis Patient,” International Wound Journal 21, no. 3 (2024): e14697, 10.1111/iwj.14697.38468432 PMC10928246

The above article, published online on 11 March 2024, in Wiley Online Library (http://onlinelibrary.wiley.com/), has been retracted by agreement between the journal Editor in Chief, Professor Keith Harding; and John Wiley & Sons Ltd. Following an investigation by the publisher, all parties have concluded that this article was accepted solely on the basis of a compromised peer review process. The editors have therefore decided to retract the article. The authors did not respond to our notice regarding the retraction.

